# Supplementary material for: The interactome of intact mitochondria by cross-linking mass spectrometry provides evidence for coexisting respiratory supercomplexes
Source: Mol Cell Proteomics. 2017 Dec 8;17(2):216–32. doi: 10.1074/mcp.RA117.000470 (PMC5795388; doi:10.1074/mcp.RA117.000470)
Supplement: Supplemental Data [file supp_RA117.000470_133922_0_supp_23978_fzffwf.zip › spectra_annotation/mito_DR_spectra_annotation/267-1-13-1-12-1.pdf]

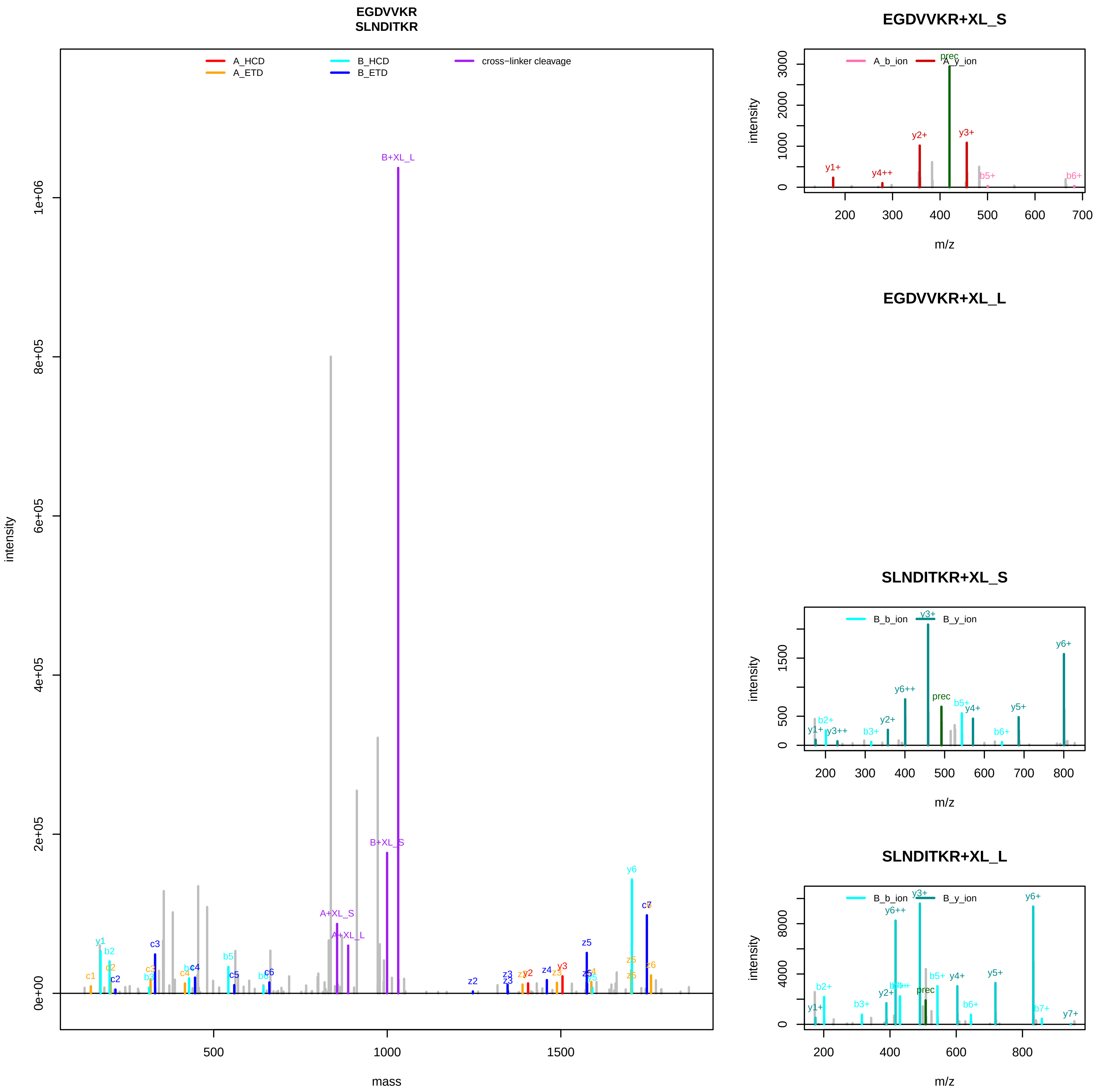
EGDVVKR
SLNDITKR
A_HCD
A_ETD
B_HCD
B_ETD
cross−linker cleavage
B+XL_L
B+XL_S
A+XL_S
A+XL_L
intensity
mass
0e+00
2e+05
4e+05
6e+05
8e+05
1e+06
500
1000
1500
y1
b2
c1
c2
c3
b3
c4
b5
c5
b6
c6
z2
z3
z4
z5
y2
y3
y6
c7
z6
EGDVVKR+XL_S
A_b_ion
A_y_ion
prec
y1+
y2+
y3+
y4++
b5+
b6+
m/z
EGDVVKR+XL_L
SLNDITKR+XL_S
B_b_ion
B_y_ion
y3+
y6+
y6++
b2+
y1+
y3++
b3+
y2+
b5+
y4+
y5+
b6+
SLNDITKR+XL_L
y7+
b7+
